# SOX7: Novel Autistic Gene Identified by Analysis of Multi-Omics Data

**DOI:** 10.21203/rs.3.rs-3346245/v1

**Published:** 2023-09-14

**Authors:** Samantha Gonzales, Jane Zizhen Zhao, Na Young Choi, Prabha Acharya, Sehoon Jeong, Moo-Yeal Lee

**Affiliations:** Florida International University; Miami Dade College Kendall Campus and School for Advanced Studies; University of North Texas; University of North Texas; Inje University; University of North Texas

**Keywords:** Genome-wide association studies (GWAS), autism spectrum disorder (ASD), gene-based association test, RNA-seq data analysis

## Abstract

**Background:**

Despite thousands of variants identified by genome-wide association studies (GWAS) to be associated with autism spectrum disorder (ASD), it is unclear which mutations are causal because most are noncoding. Consequently, reliable diagnostic biomarkers are lacking. RNA-seq analysis captures biomolecular complexity that GWAS cannot by considering transcriptomic patterns. Therefore, integrating DNA and RNA testing may reveal causal genes and useful biomarkers for ASD.

**Methods:**

We performed gene-based association studies using an adaptive test method with GWAS summary statistics from two large Psychiatric Genomics Consortium (PGC) datasets (ASD2019: 18,382 cases and 27,969 controls; ASD2017: 6,197 cases and 7,377 controls). We also investigated differential expression for genes identified with the adaptive test using an RNA-seq dataset (GSE30573: 3 cases and 3 controls) and DESeq2.

**Results:**

We identified 5 genes significantly associated with ASD in ASD2019 (KIZ-AS1, p = 8.67×10^− 10^; KIZ, p = 1.16×10^− 9^; XRN2, p = 7.73×10^− 9^; SOX7, p = 2.22×10^− 7^; LOC101929229 (also known as PINX1-DT), p = 2.14×10^− 6^). Two of the five genes were replicated in ASD2017: SOX7 (p = 0.00087) and LOC101929229 (p = 0.009), and KIZ was close to the replication boundary of replication (p = 0.06). We identified significant expression differences for SOX7 (p = 0.0017, adjusted p = 0.0085), LOC101929229 (p = 5.83×10^− 7^, adjusted p = 1.18×10^− 5^), and KIZ (p = 0.00099, adjusted p = 0.0055). SOX7 encodes a transcription factor that regulates developmental pathways, alterations in which may contribute to ASD.

**Limitations:**

The limitation of the gene-based analysis is the reliance on a reference population for estimating linkage disequilibrium between variants. The similarity of this reference population to the population of study is crucial to the accuracy of many gene-based analyses, including those performed in this study. As a result, the extent of our findings is limited to European populations, as this was our reference of choice. Future work includes a tighter integration of DNA and RNA information as well as extensions to non-European populations that have been under-researched.

**Conclusions:**

These findings suggest that SOX7 and its related SOX family genes encode transcription factors that are critical to the downregulation of the canonical Wnt/*β*-catenin signaling pathway, an important developmental signaling pathway, providing credence to the biologic plausibility of the association between gene SOX7 and autism spectrum disorder.

## Background

Autism spectrum disorder (ASD) is a heterogeneous grouping of neurodevelopmental traits that is diagnosed in approximately 1% of the world population (Fombonne, 2009). ASD conditions are characterized by attention-deficit hyperactivity disorder (ADHD), intellectual disability (ID), epilepsy, social communication deficits and restricted, repetitive, or unusual sensory-motor behaviors, or gastrointestinal problems (Gillberg et al., 2014). Extensive research efforts have gone into understanding the causes of individual differences in autistic behavior. Twin and family studies strongly demonstrate that autism has a particularly large genetic basis, with estimated heritability ranging from 40–90% (Devlin et al., 2013; Gaugler et al., 2014; Sandin et al., 2017; Tick et al., 2016). Molecular genetic studies have revealed that the genetic risk for autism is shaped by a combination of rare and common genetic variants (Consortium, 2017).

Over the past decade, genome-wide association studies (GWAS) and other types of genetic studies have identified increasing numbers of single nucleotide polymorphisms (SNPs) (Grove et al., 2019; Robinson et al., 2016) and other forms of genetic variation that are associated with ASD (Bourgeron, 2015). It has been estimated that more than 100 genes and genomic regions are associated with autism (Sanders et al., 2015; Satterstrom et al., 2020). While most of these studies focused on identifying heritable single nucleotide polymorphisms (SNPs) associated with ASD risk, other studies have demonstrated the influence of de novo mutations ranging from a single base (O’Roak et al., 2012; Sanders et al., 2012) thousands to millions of bases long (Levy et al., 2011; Sanders et al., 2011), such as copy number variants (CNVs). Several likely gene-disruptive (LGD) variants in genes such as GRIK2 (Jamain et al., 2002) and ASMT (Melke et al., 2008) affecting autism risk were found exclusively or more frequently in individuals with autism than in control groups. Additional evidence strongly suggests that mutations in NLGN3 and NLGN4 are involved in autism (Jamain et al., 2003). Additionally, deletions at Xp22.3 that include NLGN4 have been reported in several autistic individuals. Roohi et al. found that (Roohi et al., 2009) CNTN4 plays an essential role in the formation, maintenance, and plasticity of neuronal networks. Disruption of CNTN4 is known to cause developmental delay and mental retardation. This report suggests that mutations affecting CNTN4 function may be relevant to ASD pathogenesis. A review by Li and Brown (Li et al., 2012) discussed a substantial body of evidence resulting from genome-wide screening for several widely studied candidate ASD genes. Similarly, a large-scale international collaboration was conducted to combine independent genotyping data to improve statistical power and aid in robust discovery of GWS loci (Consortium, 2017). This international collaboration also identified a significant genetic correlation between schizophrenia and autism with several neurodevelopmental-related genes, such as EXT1, ASTN2, MACROD2, and HDAC4. A combined analysis investigating both rare and common gene variants supported the evidence of the role of several genes/loci associated with autism (e.g., NRXN1, ADNP, 22q11 deletion) and revealed new variants in known autism-risk genes such as ADPNP, NRXN1, NINL, and MECP2 and identified new compelling candidate genes such as KALRN, PLA2G4A, and RIMS4 (Leblond et al., 2019). Recently, Buxbaum (Buxbaum, 2022) summarized the prevalence of some genetic variants in subjects ascertained for ASD.

Research investigating the gene expression profiles of those with ASD has also proven insightful genetic contributions to ASD. The expression levels of genes containing rare mutations associated with autism were evaluated in lymphoblasts from autism cases and controls, including the aforementioned genes, such as NLGN3, NLGN4, NRXN1, and MeCP2. Of these, NLGN3 was found to be differentially expressed along with SHANK3 (Yasuda et al., 2011). More comprehensive gene expression analyses have confirmed susceptibility genes previously reported in GWAS-based analysis and identified novel differentially expressed genes and biological pathways enriched for these genes (Rahman et al., 2020). RNA sequencing data analyses have elucidated several potential drivers of autism susceptibility, such as resting-state functional brain activity (Berto et al., 2022), dopaminergic influences in the dorsal striatum (Brandenburg et al., 2020), overexpression of FOXP1, a gene involved in regulating tissue and cell type-specific gene transcription in the brain (Chien et al., 2013; Ferland et al., 2003), genome-wide alterations to lncRNA levels, downregulation of alternative splicing events, and brain region-dependent alterations in gene expression (Parikshak et al., 2016). These studies indicate that integrating GWAS and RNA-seq data analysis can provide a better picture of the various underlying mechanisms behind a heterogeneous, multifaceted condition such as ASD.

In this study, we performed whole-genome gene-based association tests for ASD with the adaptive test method (Guo & Wu, 2018) using summary statistics from two large GWAS datasets obtained from the Psychiatric Genomics Consortium (PGC). We identified 5 genes significantly associated with ASD in the ASD 2019 data. Among these 5 genes, the genes SOX7 and LOC101929229 (also known as PINX1-DT) were replicated in ASD 2017 data. Gene LOC101929229 is ncRNA. Further RNA sequencing data analysis indicated that the gene SOX7 was significantly upregulated in cases compared to controls. SOX7 encodes a member of the SOX (SRY-related HMG-box) family of transcription factors that pivotally contribute to determining cell fate and identity in many lineages. The encoded protein may function as a transcriptional regulator after forming a protein complex with other proteins, leading to autism.

## Methods

### Datasets

#### Discovery GWAS summary statistics:

The discovery dataset (labeled as asd2019) includes summary statistics from a meta-analysis of European samples derived from two cohorts: a population-based case control study from the iPSYCH project and a family trio-based study from the Psychiatric Genomics Consortium (PGC) (Grove et al., 2019). The iPSYCH samples included individuals born by a known mother who was a resident of Denmark at the time of their first birthday. Cases were identified using the Danish Psychiatric Central Research Register, using diagnoses from 2013 or earlier by psychiatrists according to diagnostic code ICD10, which includes diagnoses of childhood autism, atypical autism, Asperger’s syndrome, “other pervasive developmental disorders”, and “pervasive developmental disorder, unspecified” (Grove et al., 2019). The PGC samples consisted of 5 cohorts, whose trios were analyzed as cases and pseudo controls. Details regarding these studies can be found in (Grove et al., 2019) and (Consortium, 2017). The combined sample size consisted of 18,382 cases and 27,969 controls. Imputation and quality control were performed via PGC’s Ricopili pipeline, which ensures the production of robust, reproducible, and comparable datasets. The iPSYCH samples were processed separately in the 23 genotyping batches, while the PGC samples were processed separately for each study. Genotype imputation was performed with IMPUTE2/SHAPEIT(Bryan N. Howie, 2009; Delaneau et al., 2011) in the Ricopili pipeline using the 1000 Genomes Project phase 3 dataset as the reference set. Regions demonstrating high linkage disequilibrium were excluded, and one of the highly similar pairs of subjects identified by PLINK’s identity by state (IBS) analysis (Chang et al., 2015) was reduced at random, with a preference for retaining cases. Association was performed using PLINK on imputed dosage data, and the meta-analysis was performed using METAL (Grove et al., 2019). More detailed descriptions of each stage of the analysis can be found in Grove et al. (Grove et al., 2019). The summary statistics produced by this study and subsequently used for our analysis can be found at https://pgc.unc.edu/for-researchers/download-results/.

#### Replication GWAS summary statistics:

The replication dataset (labeled as asd2017) includes summary statistics from a European-ancestry meta-analysis performed by the Autism Spectrum Disorders Working Group (AWG) of The Psychiatric Genomics Consortium (PGC), which aimed at improving statistical power to detect loci significantly associated with ASD. The meta-analysis was performed on data from 14 independent cohorts across different ancestries totaling over 16,000 individuals. For each step in the meta-analysis, each cohort was processed individually. Individuals were excluded if they were assessed at less than 36 months of age or if diagnostic criteria were not met from the Autism Diagnostic Interview-Revised (ADI-R) or the Autism Diagnostic Observation Schedule (ADOS) domain scores. While a “worldwide” meta-analysis on this aggregate dataset was performed, we derive our replication dataset based on the smaller European-only analysis consisting of 6,197 ASD cases and 7,377 controls (Consortium, 2017). Each stage of the imputation and quality control was performed similarly to the asd2019 data: Imputation and quality control on PGC samples were performed following the PGC’s “Ricopili” pipeline. Since multiple studies were involved, necessary studies were performed to check for and remove duplicate individuals prior to imputation. Family trio-based data were organized as case and pseudo controls. Criteria for SNP retention and other pre-imputation quality control steps can be found in the study’s supplementary File 1 (Consortium, 2017). Genotype imputation was performed with IMPUTE2/SHAPEIT using the 2,184 phased haplotypes from the full 1000 Genomes Project dataset as the reference set. All 14 cohorts were tested for associations individually using an additive logistic regression model in PLINK. More detailed information about each stage of the analyses performed by this study can be found in supplementary File 1 (Consortium, 2017). The resulting summary statistics that were utilized in our analysis can be found at https://pgc.unc.edu/for-researchers/download-results/.

### Bulk RNA-Seq

The RNA dataset was obtained from a gene coexpression analysis that aimed to identify modules of coexpressed genes associated with ASD (Voineagu et al., 2011). The study can be found in the Gene Expression Omnibus (GEO) database under accession number GSE30573. Detailed descriptions of the raw data acquisition and quality control processes can be found in the supplementary information of (Voineagu et al., 2011) as well as the GEO accession viewer. Briefly, brain tissue samples (frontal cortex, temporal cortex, and cerebellum) were obtained from the Autism Tissue Project (ATP) and the Harvard Brain Bank. Cases were diagnosed using ADI-R diagnostic scores, which can be found along with other clinical data upon request from the ATP website. Total RNA was extracted from the sample tissues following the Qiagen miRNA kit instructions. Quality and concentration were assessed by an Agilent Bioanalyzer and Nanodrop, respectively. Reads were generated using an Illumina Genome Analyzer II sequencer using the manufacturer settings and were 73–76 nucleotides in length. Raw sequencing data for the frontal and temporal cortex samples were available in the SRA run selector for 3 autism cases and 3 controls (Voineagu et al., 2011).

### Quality Control & Preprocessing

#### GWAS summary data

After downloading the raw summary statistics from the PGC website, we performed quality control analysis to ensure robust and high-quality results. Only SNPs on autosomal chromosomes were used. First, SNPs with an imputation information metric (INFO) score > 0.9 were removed. Next, SNPs with strand-ambiguous alleles or nonbiallelic loci were removed as well as SNPs with duplicate rs IDs. Z scores were then calculated using each variant’s odds ratio and standard error using the equation. After quality control, the raw variants were sorted into hg19 Reference Sequence genes. Linkage disequilibrium (LD) within each gene was calculated using the 1000 Genomes European reference panel (phase 3). For each gene, a subset of the GWAS variants of the gene’s transcription start site and transcription end site were matched to the reference variants, ensuring that both used the same reference allele and flipping Z score signs if necessary. Genes that contained less than 2 SNPs were removed. Pearson’s correlation between this subset of genotypes was calculated and used as the gene-wide LD. One SNP of a pair of SNPs with perfect correlation ( within a gene was removed. The processed data were saved in 22 ‘RData’ files (one for each chromosome) containing a list of data frames, where each list element comprised 1) SNP information for a specific gene and 2) its corresponding LD matrix.

#### RNA-seq data

The sequence read archive (SRA) accession list and associated sample metadata (“SRA Run Table”) for GSE30573 were downloaded from the SRA run selector page for the study. Raw fastq files were downloaded from the SRA using the SRA Toolkit via the ‘prefetch’ and ‘fastq-dump’ commands (“SRA Toolkit,”). We used FastQC to assess the quality of reads in each file and MultiQC to visualize the results in batch format (Andrews, 2010; Ewels et al., 2016). Only 1 sample failed the ‘per sequence base quality’ assessment and was subsequently trimmed of low-quality reads using the command-line tool ‘fastq_quality_filter’ from the FastX toolkit using a minimum quality Phred score of 20 and a minimum percent of bases per read to meet that threshold of 50% (“FASTX-Toolkit,”). Reassessment via FastQC demonstrated this as sufficient trimming to meet the quality needed for downstream analysis.

#### Statistical Analysis

##### Gene-based Association Test:

To perform gene-based association testing, we used the function ‘sats’ in the R package ‘mkatr’ (Guo & Wu, 2018). This function computes p values for 3 different SNP-set testing methods using GWAS summary statistics and an LD matrix calculated from a reference panel. A brief description of each is as follows: Let m denote the number of variants considered in a gene or gene region and let $\left(z_{1},\cdots z_{m}\right)$ represent the GWAS summary statistics for each gene. Let $R=\left(r_{ij}\right)$ denote the estimated correlation between Z statistics based on variant linkage disequilibrium (LD) calculated from a reference panel (Guo & Wu, 2018). The tests included in the sats function are the sum test (a type of burden test), the squared sum test and the adaptive test. The three tests are as follows:

1. Sum test (ST): $B=\sum_{j=1}^{m}z_{j}$
2. Squared sum test (S2T): $Q=\sum_{j=1}^{m}z_{j}^{2}$
3. Adaptive test (AT): $T={min}_{\rho \in [0,1]}\;P\left(Q_{\rho }\right)$ where $Q_{\rho }=(1-\rho )Q+\rho B^{2}$ and $P\left(Q_{\rho }\right)$ denotes the p value of $Q_{\rho }$.

It can be shown that $Q_{\rho }$ asymptotically follows a weighted sum of independent chi-squared distribution with 1 degree of freedom $\left[\chi^{2}(df=1)\right]$ whose weights equal the eigenvalues of R. The minimum p value of AT is searched for over a range of ρ in the interval [0, 1] (Guo & Wu, 2018).

The ST is most valuable when all variants have the same direction of effect and approximately equal effect size, while the S2T will perform better than ST when variants have different directions of effects. AT utilizes information from both ST and S2T, meaning AT can adapt to the variants in the data better than ST or S2T alone. Indeed, the adaptive test shows the most robust performance across a wider range of scenarios (Guo & Wu, 2018). In this study, we report results based on the AT method. More details regarding the derivation of these tests and their relation to the single-variant association test can be found in (Guo & Wu, 2018).

##### Differential Gene Expression Analysis:

After passing quality control, RNA-seq reads were aligned to a reference genome using STAR (Dobin et al., 2013) by the following two steps: genome indexing and alignment to the indexed reference genome. We generated the genome index files using STAR’s – genomeGenerate flag and setting the option –sjdbOverhang to 75 to match the maximum read length across the samples. The reference genome FASTA file and corresponding annotation GTF file (GChr37/hg19, release 41) used to generate these index files were downloaded from GENCODE (Frankish et al., 2019). After alignment, we used HTSeq (Putri et al., 2022) to estimate the number of reads per gene region. For genes with gene expression counts of at least 10, we used the R package DESeq2 (Love et al., 2014) to perform differential expression analysis based on normalized gene expression counts. DESeq2 uses a generalized linear model to model the relationship between a trait and the log2-fold changes in gene expression (Love et al., 2014). We used the adjusted p value to assess significance in gene expression differential analysis.

### Computing Environment

RNA-seq quality control, alignment, and counts were processed on the lonestar6 high-performance cluster provided by TACC at the University of Texas at Austin. Differential gene expression analysis and gene-based association tests were performed in a local Linux (Windows Linux Subsystem) environment using R in RStudio (RStudio 2022.07.1 + 554).

## Results

### Gene-based Association Test

#### Discovery GWAS

**Figure 1:**
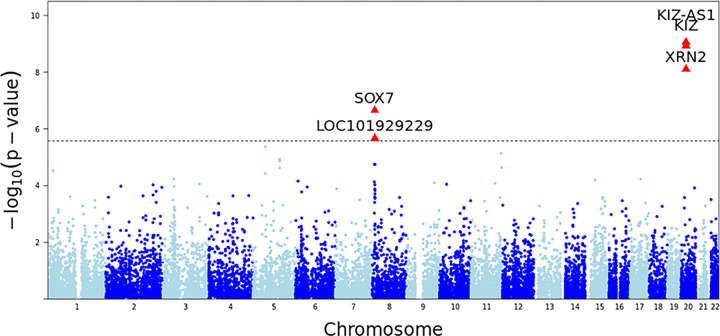
Manhattan plot of the asd2019 GWAS data. Each dot represents a gene tested for association with ASD; the dotted horizontal line represents a Bonferroni corrected p value threshold of 2.5×10^−6^.

Out of approximately 19,000 genes tested for association with ASD, 5 genes were identified as significant with Bonferroni corrected p values less than (Fig. 1 and Table 1). SOX7 () encodes a transcription factor involved in regulating embryonic development and cell fate determination (Takash et al., 2001). KIZ () encodes “Kizuna centrosomal protein”, which plays a central role in stabilizing the pericentriolar region before the spindle formation step in cellular division (Oshimori et al., 2006). A gene region that encodes a long noncoding antisense RNA for KIZ, KIZ-AS1 (), was also identified as significant; however, the function of this antisense RNA has not been determined. XRN2 () encodes a 5’–3’ exoribonuclease that is pertinent in promoting transcriptional termination (Eaton & West, 2018). Finally, LOC101929229 (), also known as PINX1-DT, is a lncRNA that is considered a “divergent transcript” of the protein coding gene PINX1. While the divergent transcript function is not defined, PINX1 encodes a protein that enables telomerase RNA binding and inhibitor activity and is involved in several related processes, including DNA biosynthesis and protein localization (Johnson, 2011).

#### Replication GWAS

Among these 5 genes, the genes SOX7 (=0.00087), LOC101929229 (=0.009), and KIZ-AS1 (=0.059) were replicated in the ASD 2017 data. KIZ (=0.06) was close to the boundary of replication in the ASD 2017 data (Table 1).

LOC101929229 is also known as PINX1-DT, which is a ncRNA gene. Significance level=2.5×10^−6^ in the analysis with the discovery dataset; 0.05 in the analysis with the replication dataset and the RNA-seq dataset. Bold highlighting indicates significance in all three (discovery, replication, and RNA-seq) analyses.

#### Differential Gene Expression Analysis

**Figure 2:**
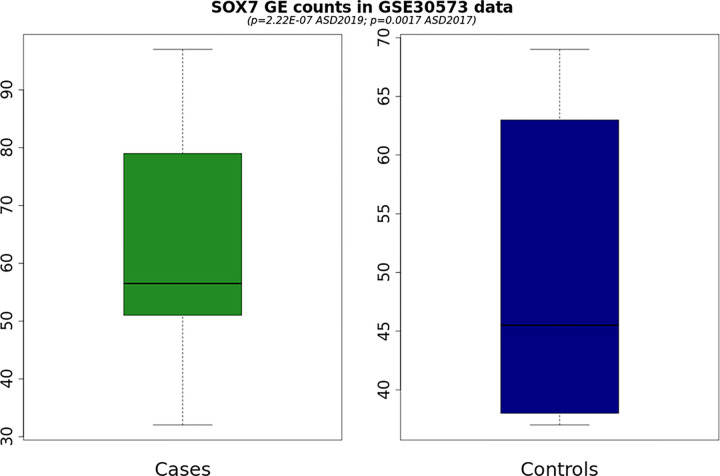
Boxplot demonstrating significant upregulation of SOX7 in autism cases (p = 0.0017).

Among the five genes identified in the discovery of GWAS, the genes SOX7 (log2FoldChange [LFC] = 1.17, ; Benjamini‒Hochberg (BH) adjusted = 0.0085), LOC101929229 (LFC=3.22, p=5.83×^−7^, adjusted p=1.18×^−5^), and KIZ (LFC=0.63, =0.00099, BH adjusted =0.0055) were also identified as significant in the differential gene expression analysis (Table 1). A comparison of case–control gene expression counts for SOX7 can be found in Figure 2, demonstrating that SOX7 is consistently upregulated in autism cases compared to controls. The expression of SOX7 is increased in autism patients relative to controls by a multiplicative factor of 2.25. In addition, the expression of LOC101929229 was increased in autism patients compared with controls by a multiplicative factor of 9.31.

## Discussion

Through powerful gene-based analysis, we identified 5 gene regions (KIZ, KIZ-AS1, XRN2, LOC101929229, and SOX7) significantly associated with ASD. Gene SOX7 and LOC101929229 (also known as PINX1-DT) were replicated by a different GWAS data (gene KIZ was close to the boundary of replication (p=0.06)) and advocated by the differential gene expression analysis performed on publicly available RNA-seq data.

KIZ is located on chromosome 20 and encodes Kizuna centrosomal protein, which aids in stabilizing the pericentriolar region of centrosomes before spindle formation. KIZ has been identified as significantly associated with autism in previous GWAS (Grove et al., 2019), TWAS (Huang et al., 2021), gene-based analysis (Alonso-Gonzalez et al., 2019), and methylation-based studies (Hannon et al., 2018), and the involvement of cell cycle regulation in autism susceptibility which has also been implicated in previous research (Packer, 2016; Pramparo et al., 2015). KIZ has also been found to be a potentially shared genetic locus between ASD and attention-deficit hyperactivity disorder (ADHD), providing support for its involvement in neurological conditions (Baranova et al., 2022).

XRN2 is located next to KIZ and encodes a 5’–3’ exonuclease that is involved in myriad RNA management processes, including transcriptional termination, miRNA expression regulation, nonsense-mediated mRNA decay, and rRNA maturation (Brannan et al., 2012; Nagarajan et al., 2013; Wang & Pestov, 2011; West et al., 2004). XRN2 has been found to play a role in regulating miRNA expression in neurons specifically, and altered miRNA expression regulation has been investigated as a potential mechanism for autism susceptibility (Abu-Elneel et al., 2008; Ghahramani Seno et al., 2011; Hicks & Middleton, 2016; Kinjo et al., 2013; Wu et al., 2016). Likewise, disruption of proper RNA metabolism as a result of altered expression of RNA binding proteins has been implicated in neurological disease as a whole, and the XRN gene family is involved in nonsense-mediated decay of mRNA, a process that has been implicated in autism pathophysiology (Marques et al., 2022; Nussbacher et al., 2019). Previous GWAS have reported SNPs in the region containing XRN2 to be significantly associated with ASD, affirmed by gene-based analysis using MAGMA (Grove et al., 2019). Additionally, a transcriptome-wide association study (TWAS) found XRN2 to be significantly upregulated in autism, in accordance with our findings (Pain et al., 2019). Another gene-based analysis found XRN2 to be associated with ASD, and upon further investigation *via* gene-network analysis and enrichment analysis, not only does XRN2 interact with several genes in the cAMP signaling pathway and RNA transport network, but the enriched KEGG/GO terms for XRN2 (spliceosome, RNA transport, and nucleic acid binding) found to be associated with ASD are also essential processes pivotal to early development (Alonso-Gonzalez et al., 2019). The extensive involvement of XRN2 in such complex mechanisms of gene expression regulation, particularly in neuronal cell types, offers possible insights into the vast heterogeneity of ASD and its overlap with other neurodevelopmental conditions. In fact, more recent research efforts have focused on ascertaining genetic commonalities between ASD and related disorders such as ADHD, obsessive compulsive disorder (OCD), and Tourette syndrome, of which XRN2 seems to be a shared significant locus (Peyre et al., 2020; Yang et al., 2021).

SOX7 is of particular interest due to its hallmark involvement in the regulation of the Wnt/ -catenin pathway, an important developmental signaling pathway. SOX7 and its related SOX family genes encode transcription factors that are critical to the downregulation of the canonical Wnt/ -catenin signaling pathway, which controls embryonic development and adult homeostasis and is involved in a multitude of cellular processes (Katoh, 2002; MacDonald et al., 2009). While the Wnt pathway is ubiquitous to nearly all tissue types, proteins involved in Wnt signaling in the brain specifically have been found to localize in the synapses and influence synaptic growth, and knockout murine models of ASD risk genes that are a part of the Wnt pathway have provided support for the disruption of this pathway in autism-like behaviors (Kwan et al., 2016). Indeed, the Wnt/ -catenin signaling pathway has been suggested as a possible avenue for autism pathogenesis in several studies (Caracci et al., 2021; de la Torre-Ubieta et al., 2016; El Khouri et al., 2021; Hormozdiari et al., 2015; Kwan et al., 2016; Quesnel-Vallières et al., 2019; Vallée et al., 2019).

SOX7 also regulates angiogenesis, vasculogenesis, and endothelial cell development, and the SOX family of transcription factors is critical to cardiovascular development (Francois et al., 2010; Kim et al., 2016). For example, SOX7 was found to be upregulated in sustained hypoxic environments, mediating angiogenesis (Klomp et al., 2020), and a knockout model of SOX7 was found to result in profound vascular defects, demonstrating that SOX7 has an essential role in vasculogenesis and angiogenesis in early development (Lilly et al., 2017). Links between the role of SOX7 in developmental delay and congenital heart disease have been investigated. Specifically, deletions in the region where SOX7 resides have been demonstrated to simultaneously cause congenital heart defects and intellectual disability (Páez et al., 2008; Wat et al., 2009).

Additionally, Wnt signaling has been demonstrated to orchestrate the differentiation of neural vasculature, such as the blood‒brain barrier (Reis & Liebner, 2013; Stenman et al., 2008). Likewise, there is evidence of vascular involvement in the development of autism (Casanova, 2007; Emanuele et al., 2010; Ouellette et al., 2020; Yao et al., 2006). One review in particular suggests that mutations affecting the delicate interactions between Wnt signaling and Shh pathways may alter blood brain barrier integrity in autism by aberrantly interacting with neurovascular molecules (Gozal et al., 2021).

Last, oxidative stress has been researched as a potential source of autism susceptibility (Bjørklund et al., 2020; Chauhan & Chauhan, 2006), and the interaction between altered vasculature and autism during oxidative stress could point to another potential source of pathogenesis (Yao et al., 2006). Indeed, the role of Wnt/ -catenin signaling in oxidative stress has been directly implicated in autism susceptibility (Zhang et al., 2012). This combination of evidence that implicates both Wnt signaling and SOX7 interactions in the multitude of interrelated processes that have been suggested as mechanisms behind the etiology of ASD, supplemented by our findings, provides ever-mounting support for more in-depth investigations of these particular genes and pathways.

Wnt/ -catenin, oxidative stress, and impaired/altered vasculature have all been implicated in the development of ASD. These three factors are involved with each other and multiple systemic processes, which may contribute to ASD symptom heterogeneity. The fact that SOX7 is involved in the regulation of Wnt/ -catenin and vasculogenesis points to a potential converging mechanism behind the pathophysiology of ASD. Additionally, the association of SOX7 with autism has been investigated directly. A case study involving a child patient exhibiting “8p23.1 duplication syndrome” revealed a de novo 1.81 Mbp duplication event on chromosome 8 (8p23.1), spanning the region where SOX7 lies (Weber et al., 2014). This patient exhibited characteristic symptoms of the duplication syndrome, including delay of motor and speech development and intellectual disability, which heavily overlap with autism and related intellectual conditions. Indeed, this patient also exhibited symptoms specific to ASD, such as repetitive compulsive behavior.

A GWAS performed in a Mexican population found that SOX7 was differentially methylated between autism cases and controls (Aspra et al., 2022). Another study also found that differential methylation was associated with an “elevated polygenic burden” for autism and further identified that two significantly associated CpG sites were located near GWAS markers for autism on chromosome 8 in the same region as SOX7 (Hannon et al., 2018). It is worth noting that this study also found evidence of SNPs associated with both autism and DNA methylation that were annotated to KIZ and XRN2, two genes that we also found to be significantly associated with ASD.

Changes in methylation lead to changes in gene expression, providing another plausible mechanism of SOX7 involvement: a change in SOX7 methylation affects the expression and thus availability of the transcription factor it encodes, which has a downstream effect on the subsequent pathways SOX7 regulates, such as Wnt/ -catenin. Indeed, both methylation studies demonstrated a negative difference in methylation between autism cases and controls. Generally, undermethylation results in a less compact 3-dimensional genome structure, allowing for greater access to the gene and an increase in expression, which we see in the higher gene expression counts in autism cases versus controls in our RNA-seq data (Figure 2) (Buitrago et al., 2021; Keshet et al., 1986; Lewis & Bird, 1991).

Finally, altered expression of SOX7 has been shown to play a role in the development of several types of gliomas. One study demonstrated that SOX7 was downregulated in human glioma, allowing cancer development through upregulated Wnt/ -catenin signaling (Zhao et al., 2016), whereas another study demonstrated that overexpression of SOX7 in high-grade glioma (HGG) promoted cancer development by promoting tumor growth via vessel abnormalization (Kim et al., 2018). These somewhat conflicting observations demonstrate that, due to its heavy involvement in regulating several intricately linked developmental and homeostatic functions, SOX7 expression must be delicately balanced. Interestingly, it has also been demonstrated that there is extensive overlap of genetic risk between autism and cancer (Crawley et al., 2016; Crespi, 2011; Gabrielli et al., 2019; Tabarés-Seisdedos & Rubenstein, 2009). SOX7 expression and its interactions may provide additional support for this conjecture, particularly due to its role in vasculature development and Wnt signaling regulation.

### Limitations

The methods performed in this study are not without limitations. Gene expression is a very dynamic process that is not only tissue dependent but also cell type specific and varies depending on the developmental stage and even external factors (Fitzgerald et al., 2004; Hsieh et al., 2000; Lawlor et al., 2017; Shen-Orr et al., 2010; Weyer & Schilling, 2003; Xu et al., 2014). Certainly, these factors affecting genetic expression mean that any autism-related genes that are differentially expressed at different developmental stages or in other varying contexts may be missed. Additionally, differential expression analysis was performed on bulk RNA, whereas it is possible that altered gene expression between autism cases and controls is cell-type specific; knowing the specifics of the expression state of specific cell types that make up key areas of the brain has a better chance of revealing mechanisms behind autism pathogenesis as well as possibly elucidating the pathophysiology behind the vast variety of ASD subtypes. Gene-based analysis also has some limitations, the most important being the reliance on a reference population for estimating linkage disequilibrium between variants. The similarity of this reference population to the population of study is crucial to the accuracy of many gene-based analyses, including those performed here. As a result, the extent of our findings is limited to European populations, as this was our reference of choice. Future work includes a tighter integration of DNA and RNA information as well as extensions to non-European populations that have been under-researched.

These limitations notwithstanding, the study has considerable strengths. The AT method used in the gene-based GWAS can not only integrate the favorable properties of sum and squared sum tests but also consider LD information among genetic variants. The heatmap of the correlation between genetic variants in SOX7 (Supplementary Figure 1) indicates that rs7005905 and rs7836366, rs10100209 and rs7836366, and rs10100209 and rs7005905 have strong positive linkage disequilibrium (LD) (ρ>0.5); rs4841432 has negative LD with other variants except for rs7009920. The strong LD in SOX7 and the powerful AT method warrant our identification of the autism-associated gene SOX7. The successful replications of SOX7 in the replication data, gene expression data, and the associated biological plausibility underscores the robustness of the finding of the connection between SOX7 and autism. This finding may significantly advance our understanding of the etiology of autism, open new opportunities to reinvigorate stalling autism drug development and increase the accuracy of risk prediction of autism, which makes early autism intervention and prevention possible.

## Conclusion

These findings suggest that SOX7 and its related SOX family genes encode transcription factors that are critical to the downregulation of the canonical Wnt/ -catenin signaling pathway, an important developmental signaling pathway, providing credence to the biologic plausibility of the association between gene SOX7 and autism spectrum disorder.

## Figures and Tables

**Table 1.** Genetic features of significant genes identified in the discovery dataset.

Gene	Chr.	Discovery (asd2019)	Replication (asd2017)	RNA-seq (GSE30573)		
		p value	p value	LFC	p value	Adj. p value
*SOX7*	8	**2.22E-07**	**0.0009**	1.17	**0.0017**	**0.0085**
*L0C101929229*	8	**2.14E-06**	**0.009**	3.22	**5.83×10** ^^**−7**^^	**1.18×10** ^**−5**^
*XRN2*	20	7.73E-09	0.10	0.34	0.001	0.007
*KIZ*	20	1.16E-09	0.06	0.63	0.001	0.006
*KIZ-AS1*	20	8.67E-10	0.059	0.062	0.43	0.58

Abbreviations: Chr.: chromosome; LFC: log2-fold change.

## Data Availability

The anonymized dataset and materials are available on reasonable request from Moo-yeal Lee (Moo-Yeal.Lee@unt.edu).

## Abbreviations

GWAS: genome-wide association studies
ASD: autism spectrum disorder
PGC: Psychiatric Genomics Consortium (PGC)
LD: linkage disequilibrium
ST: Sum test
S2T: Squared sum test
AT: Adaptive test
SRA: sequence read archive
GEO: Gene Expression Omnibus
ATP: Autism Tissue Project
SNPs: single nucleotide polymorphisms
